# Gold Miners' Phthisis

**Published:** 1902-06-21

**Authors:** 


### GOLD MINERS' PHTHISIS.

Among the newer forms of industrial diseases we must place gold miners' phthisis as met with in the deep gold mines in the Transvaal. Owing to the closing of the mines and the continuance of the war, large numbers of miners who had gone out from the colliery districts of Northumberland have returned to their old homes, and Dr. Thomas Oliver[1] says that many of these men have presented on their arrival the appearance of men broken down in health and have sooner or later succumbed to pulmonary disease. He has been able moreover to show that their lung affection has assumed the characters of a veritable pulmonary fibrosis. Gold miners' disease of the lungs is due, he says, to the inhalation of dust, and is a malady akin to stonemasons' and steel-grinders' phthisis. Like all the pneumoconioses, it is slowly developed. The earliest symptom is cough, especially in the morning, then comes shortwindedness. So long as the patient keeps quiet the breathing may be perfectly easy, but the slightest effort, or walking in the face of a wind, causes the breathing to become difficult and noisy as in asthma. By degrees the appetite is lost and emaciation becomes slowly progressive, but there is none of the night-sweating which is so characteristic of ordinary tuberculous phthisis. From first to last it is an afebrile and a painless affection. It seems to commence as a peri-bronchitis, and this is followed by an increase of the fibro-connective tissue of the lung, although, as is the case in stonemasons' phthisis, it may have a tuberculous lesion engrafted upon it. Two risks to health are incurred by the men engaged in the gold mines, besides the possible dangers which are run in the abstraction of the gold from the rock by means of what is known as the "cyanide process." One danger arises from breathing the poisonous gases which result from the large quantities of high explosives used in sinking the shafts, and from the other impurities which accumulate in the air at the bottom of these deep shafts owing to the difficulty of ventilating them. But it is in the act of mining that the danger of pulmonary disease arises. Gold mining is a very dusty process, the rock is hard and has to be drilled by machinery, and it is the dry fine dust given off in drilling the rock that is the cause of lung trouble in gold miners. A man may work five or six years before he is rendered *hors de combat* by the dust. Gold miners' phthisis therefore kills men when they are still comparatively young, usually before they reach the age of forty years.

[1] Lancet, June 14.

